# MicroRNA panels as diagnostic biomarkers for colorectal cancer: A systematic review and meta-analysis

**DOI:** 10.3389/fmed.2022.915226

**Published:** 2022-11-07

**Authors:** Daniel Sur, Shailesh Advani, Dejana Braithwaite

**Affiliations:** ^1^Department of Medical Oncology, The Oncology Institute “Prof. Dr. Ion Chiricuţă” Cluj-Napoca, Cluj-Napoca, Romania; ^2^11th Department of Medical Oncology, University of Medicine and Pharmacy “Iuliu Haţieganu”, Cluj-Napoca, Romania; ^3^Department of Oncology, Georgetown University School of Medicine, Washington, DC, United States; ^4^Terasaki Institute of Biomedical Innovation, Los Angeles, CA, United States; ^5^Department of Epidemiology, University of Florida College of Public Health and Health Professions, Gainesville, FL, United States; ^6^University of Florida Health Cancer Center, Gainesville, FL, United States; ^7^Department of Aging and Geriatric Research, University of Florida College of Medicine, Gainesville, FL, United States

**Keywords:** miRNA, panels, colorectal cancer, diagnostic, biomarker

## Abstract

**Background:**

Circulating microRNAs (miRNA) have emerged as promising diagnostic biomarkers for several diseases, including cancer. However, the diagnostic accuracy of miRNA panels in colorectal cancer (CRC) remains inconsistent and there is still lack of meta-analyses to determine whether miRNA panels can serve as robust biomarkers for CRC diagnosis.

**Methods:**

This study performed a systematic review and meta-analysis to evaluate the clinical utility of miRNA panels as potential biomarkers for the diagnosis of CRC. The investigation systematically searched PubMed, Medline, Web of Science, Cochrane Library, and Google Scholar (21-year span, between 2000 and 2021) to retrieve articles reporting the diagnostic role of miRNA panels in detecting CRC. Diagnostic meta-analysis of miRNA panels used diverse evaluation indicators, including sensitivity, specificity, Positive Likelihood Ratio (PLR), Negative Likelihood Ratio (NLR), Diagnostic Odds Ratio (DOR), and the area under the curve (AUC) values.

**Results:**

Among the 313 articles identified, 20 studies met the inclusion criteria. The pooled estimates of miRNA panels for the diagnosis of CRC were 0.85 (95% CI: 0.84–0.86), 0.79 (95% CI: 0.78–0.80), 4.06 (95% CI: 3.89–4.23), 0.20 (95% CI: 0.19–0.20), 22.50 (95% CI: 20.81–24.32) for sensitivity, specificity, PLR, NLR, and DOR, respectively. Moreover, the summary receiver operating characteristics (SROC) curve revealed an AUC value of 0.915 (95% CI: 0.914–0.916), suggesting an outstanding diagnostic accuracy for overall miRNA panels. Subgroup and meta-regression analyses demonstrated that miRNA panels have the highest diagnostic accuracy within serum samples, rather than in other sample-types – with a sensitivity, specificity, PLR, NLR, DOR, and AUC of 0.87, 0.86, 7.33, 0.13, 55.29, and 0.943, respectively. Sensitivity analysis revealed that DOR values did not differ markedly, which indicates that the meta-analysis had strong reliability. Furthermore, this study demonstrated no proof of publication bias for DOR values analyzed using Egger’s regression test (*P* > 0.05) and funnel plot. Interestingly, miR-15b, miR-21 and miR-31 presented the best diagnostic accuracy values for CRC with sensitivity, specificity, PLR, NLR, DOR, and AUC values of 0.95, 0.94, 17.19, 0.05, 324.81, and 0.948, respectively.

**Conclusion:**

This study’s findings indicated that miRNA panels, particularly serum-derived miRNA panels, can serve as powerful and promising biomarkers for early CRC screening.

**Systematic review registration:**

[www.crd.york.ac.uk/prospero], identifier [CRD42021268172].

## Introduction

Colorectal cancer (CRC) is the second-leading cause of cancer-related mortalities and the third most prevalent cancer globally in 2020, with an expected 935,000 deaths and 1.93 million cases globally (1). Surgery, chemotherapy, targeted therapy, immunotherapy, and radiation are among of the most widely used CRC treatment options (2). CRC can be cured during an initial stage if diagnosed early. People with stage I/II colorectal cancer, stage III/IVA colorectal cancer, and stage IVB/IVC colorectal cancer have an estimated 5-year survival rate of 90, 72, and 14% respectively (3). In high-risk Stage II and Stage III colon cancer patients, postoperative 5-fluorouracil-based chemotherapy remains a benchmarked standard of care. Combination chemotherapy, differing novel systemic and regional multimodality therapies, metastasectomies, and other local treatments, such as hyperthermic intraperitoneal chemotherapy, are all emerging therapies for metastatic CRC (3, 4). However, even though screening techniques such as colonoscopy, fecal-based diagnostic and plasma-based assays for early identification are available, their diagnostic use is limited owing to prohibitive costings and low patient compliance (5–10). Consequently, there is an urgent need for novel diagnostic and prognostic biomarkers for the early diagnosis of CRC. Furthermore, the commonly utilized serum tumor biomarkers carcinoembryonic antibody (CEA), carbohydrate antibody 19-9 (CA19-9), are neither very sensitive nor specific (11).

MicroRNAs (miRNAs) are small, non-coding RNA molecules that regulate the expression of genes complementary binding onto the 3’ untranslated regions of target mRNAs. MiRNAs regulate a wide range of biological activities, including cell cycle, differentiation, proliferation, apoptosis, stress tolerance, energy metabolism, and immune response (12). MiRNAs were identified as stable biomarkers in body fluids such as plasma, saliva, urine, and feces (13). Since the first report in 2002 on the downregulation of miR-15 and miR-16 for chronic lymphocytic leukemia (14), extensive data revealed that expression of miRNAs is highly dysregulated in the development and progression of several types of cancers (brain, lung, breast, liver, and prostate) (15–21). More than 2,000 different microRNAs were revealed over the past few years, and they seem to contribute to the regulatation of 30% of the human genome (22). An increasing number of microRNAs are known to be deregulated in CRC and are potential biomarker candidates (23). In CRC, proliferation, migration, and invasion features were shown to be inhibited or increased by MiR-18a, miR-155, miR-205-5p or miR-494, miR-598, miR-17-3p, respectively. In addition, MiR-106a and miR-7 have been linked to apoptosis or even resistence to programmed cell death. In CRC cells, miR-221 and miR-214 inhibit autophagy. MiR-192/215 and miR-19b-1 are transcription factors that regulate metabolic pathways (24).

However, one of the biggest barriers to employ miRNAs as a diagnostic tool is the varying levels of miRNAs within individuals having differing tumor types.

Case in point, patients suffering from cancers such as colorectal, lung, breast, prostate, liver, esophageal, and endometrial cancers had upregulated serum levels of miR-21 – one of the most researched miRNAs in human malignancies (25). However, other studies revealed that miR-26a and miR-30a were downregulated in breast cancer, hepatocellular carcinoma and renal cancer (26). Consequently, it has been proposed that a panel of selected miRNAs, which includes tissue-specific miRNAs, could have increased target-organ specificity and diagnostic utility than a single miRNA/well-established clinical biomarker. Lin et al. highlighted an increased sensitivity of a blood miRNA classifier panel, including seven miRNAs – miR-29a, miR-29c, miR-133a, miR-143, miR-145, miR-192, and miR-505, to identify hepatocellular carcinoma, particularly early-stage, than á-fetoprotein (27). Even though several studies demonstrated the potential of miRNAs as biomarkers, reports comparing the robustness of different panels of miRNAs panels for CRC diagnosis are limited.

Hence, the aim of this systematic review and meta-analysis was to assess the clinical utility of miRNA panels as diagnostic biomarkers for CRC.

## Methods

### Search strategy

This systematic review and meta-analysis were carried out according to the Preferred Reporting Items for Systematic Reviews and Meta-Analyses (PRISMA) guidelines (28). This systematic review was registered to PROPERO (Registration ID: CRD42021268172). A comprehensive search was performed across PubMed, Medline, Cochrane, and Google Scholar databases (spanning a 21-year period between 2000 and 2021) by two independent authors (DS; SA) to identify potentially eligible articles. This was performed through a combination of keywords and Medical Subject Headings (MeSH): ((“MicroRNAs”[Mesh]) OR (((((((((((((((((MicroRNA [Title/Abstract]) OR miRNAs[Title/Abstract]) OR Micro RNA[Title/Abstract]) OR RNA, Micro[Title/Abstract]) OR miRNA[Title/Abstract]) OR Primary MicroRNA[Title/Abstract]) OR MicroRNA, Primary [Title/Abstract]) OR Primary miRNA[Title/Abstract]) OR miRNA, Primary[Title/Abstract]) OR pri-miRNA[Title/Abstract]) OR pri miRNA[Title/Abstract]) OR RNA, Small Temporal[Title/Abstract]) OR Temporal RNA, Small[Title/Abstract]) OR stRNA [Title/Abstract]) OR Small Temporal RNA[Title/Abstract]) OR pre-miRNA[Title/Abstract]) OR pre miRNA[Title/Abstract])) AND ((“Colorectal Neoplasm”[Mesh]) OR (((Colorectal Tumor [Title/Abstract]) OR rectal Neoplasm [Title/Abstract]) OR rectal Tumor [Title/Abstract])) AND ((“Diagnosis”[Mesh]) OR ((Diagnoses [Title/Abstract]) OR Diagnoses and Examinations [Title/Abstract])).

The cross-references from selected studies were further searched for additional articles. Articles identified through forward/backward search were screened and evaluated using identical study selection criteria.

#### Study selection criteria

Relevant articles were screened by title and abstract upon removing duplicates. Studies were eligible for inclusion if they described adult patients (aged 18 years or above) having colon, rectal and colorectal cancer mentioned, with at least one miRNA-based panel measured in a biological specimen. The selected studies were consequently examined in full-text to confirm eligibility.

#### Inclusion criteria

Inclusion criteria for articles were: (i) studies involving miRNA expression among CRC patients and control groups; (ii) studies involving clinical patient data; (iii) studies that reported miRNA profiling platforms or panels, not single miRNAs; (iv) publications reporting sensitivity, specificity, and area under the ROC curve (AUC) outcomes; and (v) studies published in English language.

#### Exclusion criteria

Exclusion criteria for articles were: (i) no full-text electronically available; (ii) publication in a language other than English; (iii) comments, letters, editorials, protocols, guidelines, case reports and review articles; (iv) *in vitro* or preclinical studies; and (v) studies with insufficient outcome data.

### Data extraction

Two independent authors (DS; SA) retrieved information from the eligible articles following the inclusion and exclusion criteria, and data were collected on a standardized data sheet that included: first author name and publication year, country, ethnicity, TNM stage, biological specimen, gender, sample size, miRNAs expression, measurement method, and the outcomes of interest: sensitivity, specificity and AUC. The results of both independent reviewers were compared, and disagreements on search strategy, article inclusion and data extraction were resolved by an independent reader as a tiebreaker.

## Study quality assessment

The methodologic quality of the included studies was evaluated independently, by two authors, using the Quality Assessment of Diagnostic Accuracy Studies-2 (QUADAS-2) tool, which included four criteria: “patient selection,” “index test,” “reference standard,” and “flow and timing” and judged bias and applicability (29). Each was assessed in terms of risk of bias, and the first three domains were assessed with respect to applicability. Each item was answered with “yes,” “no,” or “unclear.” The answer of “yes” meant low risk of bias, whereas “no” or “unclear” meant the opposite. Any disagreements were resolved by inviting a third reviewer (DB) to participate in the discussion.

## Statistical analysis

Diagnostic meta-analysis of miRNA panels was conducted on the analytical software Meta-disc 1.4 and the statistical software RevMan Version 5.4 (Cochrane Collaboration, Oxford, United Kingdom) in order to analyze the pooled sensitivity and specificity with 95% confidence intervals (CIs) across studies. The data were considered statistically significant when two-sided *P* < 0.05. Due to variations in the basic features of included articles, their diverging results could have been caused by heterogeneity or random error. Therefore, the Cochrane chi-squared test was used to evaluate heterogeneity among articles, with *P*-value < 0.05 indicating the existence of heterogeneity. To estimate the impact of heterogeneity on the meta-analysis, *I*^2^ value was also calculated. If *P* < 0.05 and *I*^2^ > 50%, heterogeneity was defined as significant. The summary receiver operating characteristic (SROC) curve and the area under the curve (AUC) were also used, based upon the sensitivity and specificity of each study to assess diagnostic performance. Subgroup and meta-regression analyses were performed to identify potential sources of heterogeneity, according to the characteristics of the included studies and using several common evaluation indicators, including sensitivity, specificity, Positive Likelihood Ratio (PLR), Negative Likelihood Ratio (NLR), Diagnostic Odds Ratio (DOR), and AUC value. Sensitivity analysis was also carried out to evaluate heterogeneity. Finally, Egger’s test was conducted via Statistical Package for Social Sciences (SPSS^®^) version 25 to evaluate publication bias. This was further assessed by visual inspection of symmetry within funnel plots.

## Results

### Study selection

**FIGURE 1:**
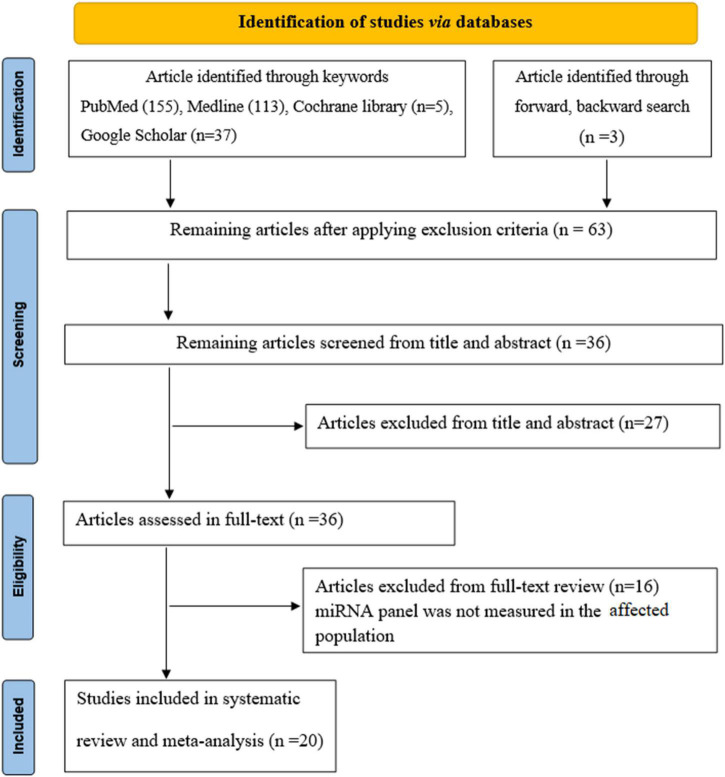
Flowchart of the literature study process and selection.

The flowchart represents the search and selection strategy for the study. The initial search resulted in a total of 313 studies, consisting of PubMed (*n* = 155), Medline (*n* = 113), Cochrane Library (*n* = 5), Google Scholar (*n* = 37) articles, together with articles identified through forward/backward search (*n* = 3). After applying the exclusion criteria, a total of 250 studies were excluded, resulting in 63 articles selected for further evaluation. Furthermore, 27 articles were excluded because of title and abstract screening criteria. The full-text of the remaining 36 articles was reviewed. In addition, 16 articles were excluded from full-text review and finally 20 studies were considered for this meta-analysis (Figure 1).

#### Characteristics of the selected studies

The 20 included studies presented an observational design. They were published between 2010 and 2021 and distributed among seven countries. The majority of studies were conducted in China (30–41) (*n* = 12) followed by Spain (42, 43) and USA (44, 45) (*n* = 2 for each one). However, Republic of Korea (46), Poland (47), Czech Republic (48), and Sweden (49) reported only one study.

In this systematic review and meta-analysis, the sample size of the included articles varied from 43 to 703 participants, with 3,339 cancer cases and 2,468 control cases, in total.

miRNAs were measured from plasma, serum, and stool within twelve (30, 31, 34, 37–42, 44, 47, 49), five (32, 33, 35, 36, 48) and two (30, 46) articles, respectively. However, only one study detected miRNAs in whole blood (45), one in tissue (45) and one in saliva (43) as specimens, respectively. Quantitative real-time polymerase chain reaction (RT-qPCR) assay was used in all studies to detect the expression levels of miRNAs, while sequencing was used in only one study. Among the 20 included articles, 7 and 6 studies reported a panel of two (27, 30, 34, 41, 44, 46, 47) and three (31, 33, 36–38, 45) miRNAs. Three studies described a panel of four miRNAs (35, 48, 49) while two studies cited a panel of five miRNAs (32, 43). However, a panel of six (42) and seven miRNAs (39) was described by one study each. Regarding miRNA expression, fifteen (30, 34–36, 38–40, 42–49) out of twenty studies highlighted an upregulation of miRNA expression in plasma, serum, stool, saliva, whole blood, or tissue specimens among CRC patients, while five studies (31, 36–38, 41) revealed a downregulation of miRNA expression in plasma and serum specimens among CRC patients.

The sensitivity of miRNAs panels varied between 0.66 and 0.96, while specificity ranged between 0.37 and 0.95. The area under the curve (AUC) varied between 0.751 and 0.960.

Studies’ features are recapitulated in Table 1.

**TABLE 1:** Characteristics of the included studies.

Article	Country	Ethnicity	TNM stage (I-IV)	Biological specimen (*n*)	Sample size: *n*	Gender: *n*	miRNA panel	Expression	Measurement method	Sensitivity	Specificity	AUC
Chang et al. (30)	China	Asian	Stage I Stage II Stage III Stage IV	Stool (447)	CRC:138 HC:309	Male: 277 Female: 170	miRNA-223 miRNA-92a	Up	qRT-PCR	0.96	0.75	0.907
				Plasma (398)	CRC:215 HC:183	Male: 231 Female: 167	miRNA-223 miRNA-92a					
Choi et al. (46)	Republic of Korea	Asian	Stage I Stage II Stage III Stage IV	Stool (58)	CRC:29 HC:29	Male: 34 Female: 24	miR-92a miR-144*	Up	qRT-PCR	0.96	0.37	0.673
Fang et al. (31)	China	Asian	Stage I Stage II Stage III Stage IV	Plasma (353)	CRC:223 HC:130	Male: 59 Female: 52	miR-24 miR-320a miR-423-5p	Down	qRT-PCR	0.90	0.70	0.941
Guo et al. (32)	China	Asian	Stage I Stage II Stage III Stage IV	Serum (575)	CRC:217 CRA:168 HC:190	Male: 362 Female: 213	miRNA-1246 miRNA-202-3p miRNA-21-3p miRNA-1229-3p miRNA-532-3p	ND	qRT-PCR	0.91	0.91	0.960
Han et al. (33)	China	Asian	ND	Serum (390)	CRC:123 CRA: 117 HC:150	Male: 194 Female: 196	miR-15b miR-21 miR-31	ND	qRT-PCR	0.95	0.94	0.948
Herreros-Villanueva et al. (42)	Spain	Caucasian	Stage I Stage II Stage III Stage IV	Plasma (397)	CRC:297 HC:100	Male: 174 Female: 123	miRNA19a miRNA19b miRNA15b miRNA29a miRNA335 miRNA18a	Up	qRT-PCR	0.85	0.90	0.920
Huang et al. (34)	China	Asian	Stage I Stage II Stage III Stage IV	Plasma (196)	CRC:137 HC:59	Male: 100 Female: 96	miR-29a miR-92a	Up	qRT-PCR	0.83	0.84	0.883
Jin et al. (35)	China	Asian	Stage III Stage IV	Serum (43)	CRC: 25 HC: 18	Male: 15 Female:10	miR-21-5p miR-1246 miR-1229-5p miR-96-5p	Up	qRT-PCR	0.78	0.88	0.804
Kanaan et al. (44)	USA	Caucasian	Stage I Stage II Stage III Stage IV	Plasma (71)	CRC: 45 HC: 26	Male: 39 Female:32	miR-431 miR-139-3p	Up	qRT-PCR	0.91	0.57	0.829
Krawczyk et al. (47)	Poland	Caucasian	Stage I Stage II	Plasma (124)	CRC: 54 HC: 70	Male: 76 Female:48	miR-506 miR-4316	Up	qRT-PCR	0.75	0.63	0.751
Liu et al. (40)	China	Asian	Stage I Stage II Stage III Stage IV	Plasma (449)	CRC:308 HC: 141	Male: 299 Female:150	miR-27a miR-130a	Up	qRT-PCR	0.85	0.90	0.899
Peng et al. (36)	China	Asian	Stage I Stage II Stage III Stage IV	Serum (232)	CRC:112 HC: 120	Male: 121 Female: 111	miR-30e-3p, miR-146a-5p	Up	qRT-PCR	0.80	0.78	0.883
							miR-148a-3p	Down				
Rapado-González et al. (43)	Spain	Caucasian	Stage I Stage II Stage III Stage IV	Saliva (112)	CRC:65 HC: 47	Male: 65 Female: 47	miR-186-5p miR-29a-3p miR-29c-3p miR-766-3p miR-491-5p	Up	qRT-PCR	0.72	0.66	0.754
Tan et al. (37)	China	Asian	Stage I Stage II Stage III Stage IV	Plasma (235)	CRC:101 HC:134	Male: 118 Female: 117	miR-144-3p miR-425-5p miR-1260b	Down	qRT-PCR	0.93	0.91	0.954
Vychytilova-Faltejskova et al. (48)	Czech Republic	Caucasian	Stage I Stage II Stage III Stage IV	Serum (703)	CRC:427 HC:276	Male: 379 Female: 324	miR-23a-3p miR-27a-3p miR-142-5p miR-376c-3p	Up	qRT-PCR	0.89	0.81	0.917
Wang et al. (41)	China	Asian	Stage I Stage II Stage III Stage IV	Plasma (191)	CRC:133 HC:58	Male: 98 Female: 93	miR-601 miR-760	Down	qRT-PCR	0.83	0.69	0.792
Wang et al. (38)	China	Asian	Stage I Stage II Stage III Stage IV	Plasma (241)	CRC:124 HC:117	Male: 119 Female:122	miR-7 miR-93	Down	qRT-PCR	0.82	0.89	0.897
							miR-409-3p	Up				
Wikberg et al. (49)	Sweden	Caucasian	Stage I Stage II Stage III Stage IV	Plasma (201)	CRC:67 HC:134	Male: 99 Female:102	miR-18a miR-21 miR-22 miR-25	Up	qRT-PCR	0.81	0.80	0.930
Wu et al. (45)	USA	Caucasian	Stage I Stage II Stage III Stage IV	Whole blood (25)	HC:25	Male: 12 Female:13	hsa-miR-451a hsa-miR-144-5p hsa-miR-200b-3p	Up	qRT-PCR Sequencing	0.66	0.95	0.890
				Tissue (95)	CRC:75 HC:20	Male: 19 Female:56						
Zhang et al. (39)	China	Asian	Stage I Stage II Stage III Stage IV	Plasma (271)	CRC:139 HC:132	Male: 158 Female:113	miR-103a-3p miR-127-3p miR-151a-5p miR-17-5p miR-181a-5p miR-18a-5p miR-18b-5p	Up	qRT-PCR	0.76	0.86	0.895

CRC, colorectal cancer; CRA, colorectal adenoma; HC, healthy control; ND, not defined.

## Study quality assessment

**FIGURE 2:**
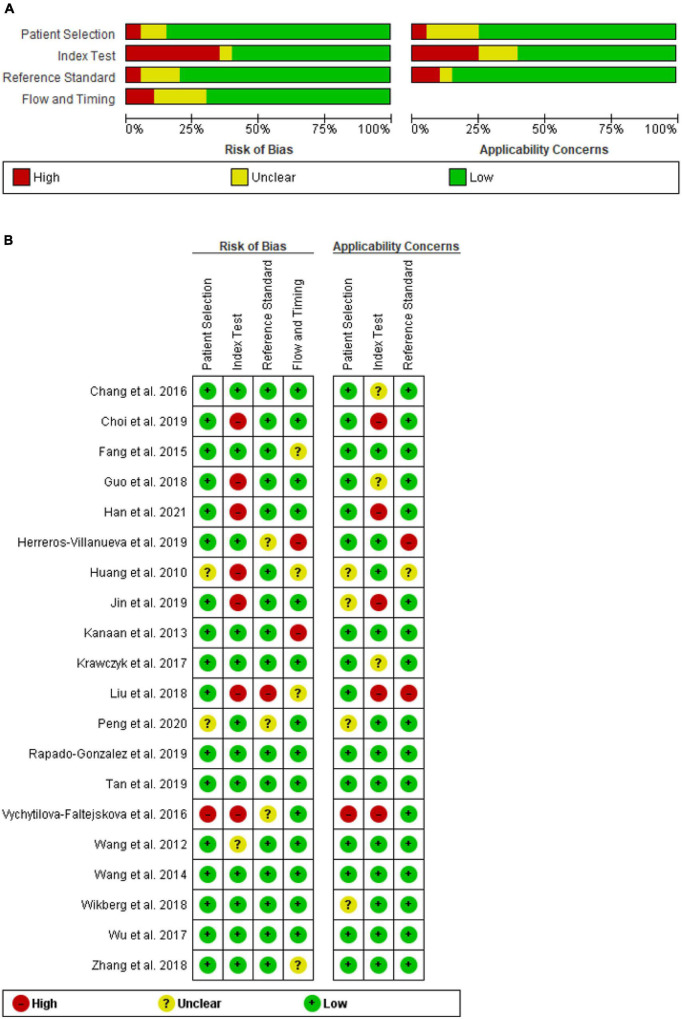
QUADAS-2 assessment of studies in terms of risk of bias and applicability concerns’ **(A)** graph and **(B)** summary.

The quality of the 20 studies was methodologically assessed using QUADAS-2 tool. Patient selection plays such a role in conducting experiments, that data used in this meta-analysis were mainly from validated groups. Overall, the qualities of included studies were satisfying and eligible. This study revealed a high-risk bias and applicability concerns, mainly concentrated on the field of index test, due to presetting the threshold. Figure 2 highlights details of the quality assessment form.

### Diagnosis accuracy of microRNA panels in colorectal cancer

**FIGURE 3:**
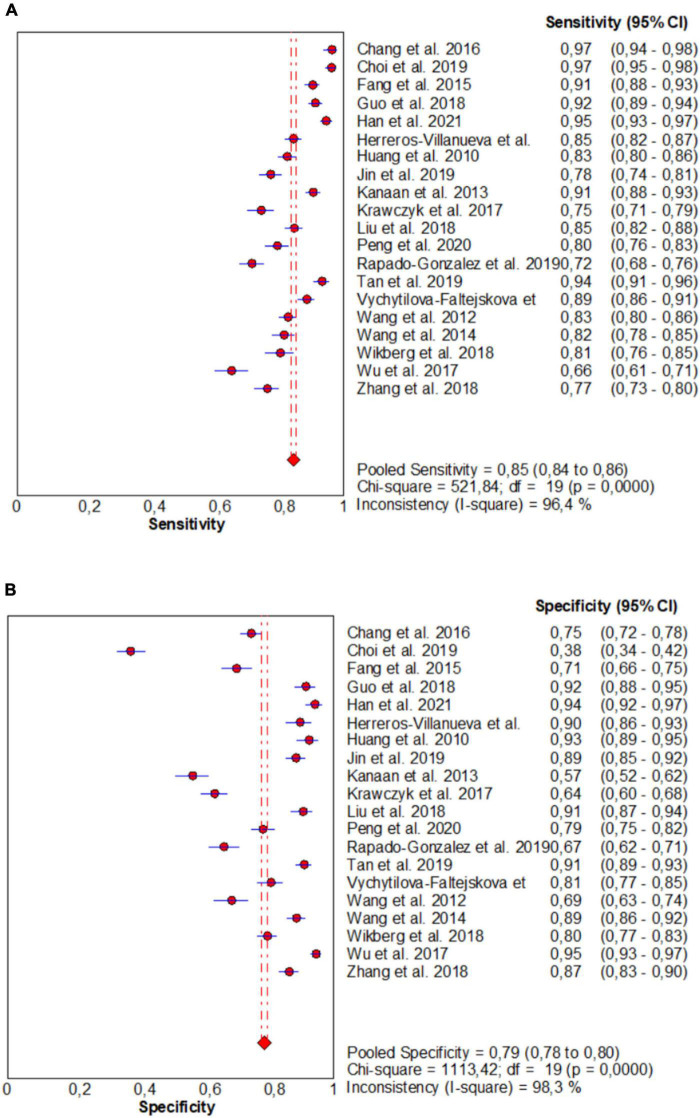
Forest plots for studies on overall microRNAs (miRNAs) used in the diagnosis of CRC among 20 studies included in the present meta-analysis. **(A)** Sensitivity; **(B)** specificity.

**FIGURE 4:**
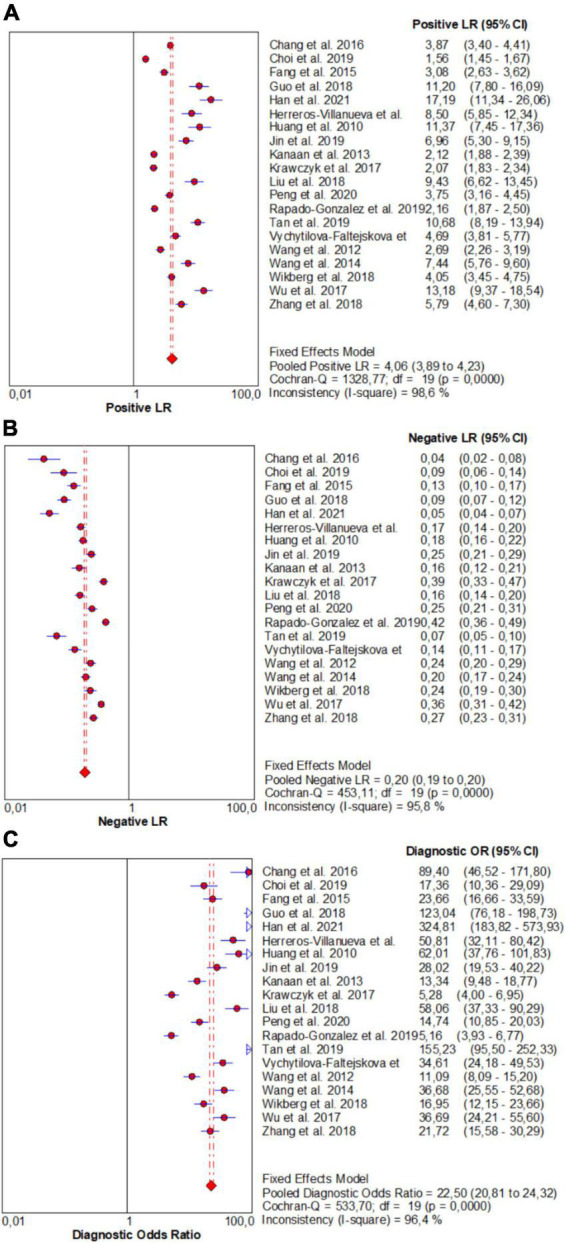
Forest plots for studies on overall microRNAs (miRNAs) used in the diagnosis of CRC among 20 studies included in the present meta-analysis. **(A)** PLR; **(B)** NLR; **(C)** DOR.

From forest plots of pooled data (20 studies), this study found significant heterogeneity in sensitivity (Chi^2^ = 521.84, *P* = 0.00, *I*^2^ = 96.4%), specificity (Chi^2^ = 1113.42, *P* = 0.00, *I*^2^ = 98.3%), PLR (Cochran-Q = 1328.77, *P* = 0.00, *I*^2^ = 98.6%), NLR (Cochran-Q = 453.11, *P* = 0.00, *I*^2^ = 95.8%), and DOR (Cochran-Q = 533.70, *P* = 0.00, *I*^2^ = 96.4%) outcomes (Figures 3 and 4). Consequently, the random-effect model was used to calculate pooled estimates.

The pooled estimate of overall miRNA for diagnosis of CRC were 0.85 (95%CI: 0.84–0.86) for sensitivity and 0.79 (95%CI: 0.78–0.80) for specificity (Figure 3).

Similarly, the pooled estimate of overall miRNA panels for diagnosis of CRC were as follows: PLR, 4.06 (95% CI: 3.89–4.23); NLR, 0.20 (95% CI: 0.19–0.20); and DOR, 22.50 (95% CI: 20.81–24.32) (Figure 4).

**FIGURE 5:**
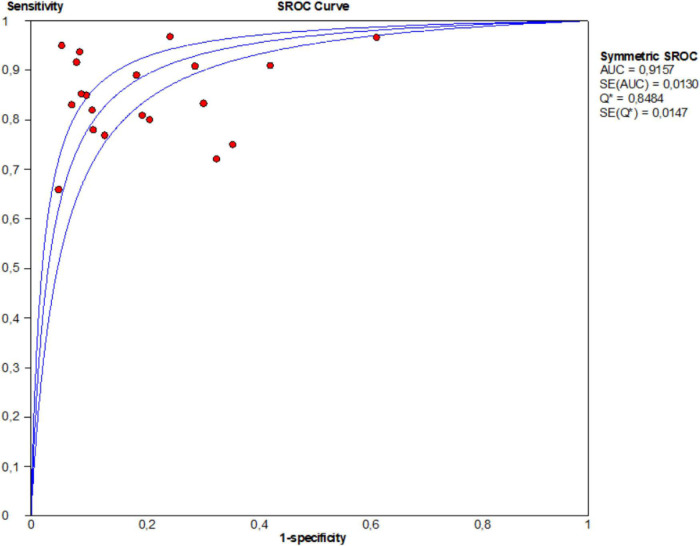
Summary receiver operator characteristic (SROC) curves based on microRNA panels of the included studies.

Moreover, this study plotted the SROC curve to evaluate diagnostic accuracy (Figure 5). AUC was 0.915 (95% CI: 0.914–0.916), suggesting an outstanding diagnostic accuracy of overall miRNA panels.

### Comparison of microRNA panels

Comparing miRNA panels, this study revealed that the panel described by Han et al. (33) (miR-15b, miR-21, miR-31) presented the best diagnostic accuracy values for CRC: sensitivity, specificity, PLR, NLR, DOR, and AUC of 0.95, 0.94, 17.19, 0.05, 324.81, and 0.948, respectively. However, the panel cited by Krawczyk et al. (47) (miR-506, miR-4316) demonstrated the lowest diagnostic accuracy values for CRC: sensitivity, specificity, PLR, NLR, DOR, and AUC of 0.75, 0.63, 2.07, 0.39, 5.28, and 0.751, respectively.

### Subgroup and meta-regression analyses

As significant heterogeneity (*P* < 0.05) was found in all parameters of diagnostic performance (sensitivity, specificity, PLR, NLR, DOR, and AUC), in addition to using the random effects model to calculate the pool estimates, this investigation further conducted meta-regression and subgroup analyses to explore between-study heterogeneity and to identify the potential sources of heterogeneity by exploring study characteristics, which include country, ethnicity, and biological specimen (Table 2). The sensitivity, specificity, PLR, NLR, DOR, and AUC values were significantly influenced by country, ethnicity and biological samples, which indicated that they were a source of heterogeneity (*P* < 0.05).

**TABLE 2:** Subgroup and meta-regression analyses of diagnostic accuracy of miRNA panels for diagnosis of CRC.

miRNAs	Sensitivity (95%CI)	Specificity (95%CI)	PLR (95%CI)	NLR (95%CI)	DOR (95%CI)	AUC (95%CI)
Overall	0.85 (0.84–0.86)	79% (0.78–0.80)	4.06 (3.89–4.23)	0.20 (0.19–0.20)	22.50 (20.81–24.32)	0.9157 (0.915–0.917)
**Country** China Spain USA Republic of Korea Poland Czech Republic Sweden P	0.86 (0.85–0.87) 0.79 (0.77–0.81) 0.82 (0.79–0.84) 0.96 (0.94–0.98) 0.75 (0.70–0.79) 0.89 (0.86–0.91) 0.80 (0.76–0.85) 0.01	0.84 (0.83–0.85) 0.75 (0.71–0.78) 0.81 (0.78–0.83) 0.37 (0.33–0.42) 0.63 (0.59–0.67) 0.81 (0.76–0.84) 0.80 (0.76–0.83) 0.009	6.54 (4.72–9.07) 4.24 (0.91–19.80) 5.24 (0.76–35.68) 1.55 (1.45–1.67) 2.06 (1.83–2.33) 4.69 (3.81–5.77) 4.04 (3.44–4.74) 0.006	0.14 (0.11–0.19) 0.26 (0.10–0.65) 0.24 (0.10–0.53) 0.09 (0.05–0.14) 0.39 (0.33–0.46) 0.13 (0.10–0.17) 0.23 (0.19–0.29) 0.007	47.52 (27.79–81.27) 16.06 (1.69–152.44) 21.96 (8.14–59.20) 17.35 (10.35–10.35) 5.27 (4.00–6.95) 34.60 (24.18–49.52) 16.95 (12.14–23.66) 0.001	0.936 (0.930–0.939) 0.837 (0.830–0.841) 0.859 (0.851–0.862) 0.673 (0.671–0.678) 0.751 (0.748–0.756) 0.917 (0.910–0.920) 0.930 (0.928–0.932) 0.005
**Ethnicity** Caucasian Asian P	0.81 (0.80–0.82) 0.86 (0.86–0.87) 0.01	0.76 (0.75–0.78) 0.80 (0.79–0.81) 0.01	4.01 (2.66–6.05) 5.91 (3.56–9.81) 0.006	0.24 (0.17–0.34) 0.13 (0.10–0.18) 0.004	16.73 (8.4–33.35) 44.01 (26.50–73.10) 0.001	0.876 (0.870–0.880) 0.936 (0.931–0.938) 0.001
**Biological specimen** Plasma Serum Stool Saliva Whole blood/Tissue P	0.84 (0.83–0.85) 0.87 (0.86–0.88) 0.96 (0.95–0.97) 0.72 (0.68–0.75) 0.65 (0.60–0.70) 0.009	0.79 (0.78–0.80) 0.86 (0.85–0.88) 0.59 (0.56–0.62) 0.66 (0.61–0.71) 0.86 (0.83–0.89) 0.008	5.01 (3.43–7.32) 7.33 (4.36–12.30) 2.45 (0.96–6.23) 2.16 (1.87–2.49) 13.17 (9.36–18.35) 0.002	0.18 (0.15–0.23) 0.13 (0.08–0.23) 0.06 (0.03–0.13) 0.41 (0.36–0.48) 0.35 (0.30–0.41) 0.001	27.21 (15.71–47–12) 55.29 (21.23–143.95) 38.89 (7.75–195.22) 5.16 (3.93–6.77) 36.69 (24.21–55.6) 0.001	0.876 (0.872–0.879) 0.943 (0.940–0.947) 0.790 (0.786–0.793) 0.754 (0.751–0.759) 0.890 (0.887–0.895) 0.001

Regarding country, miRNA panels had the highest overall diagnostic accuracy in China, with sensitivity of 0.86, specificity of 0.84, PLR of 6.54, NLR of 0.14, DOR of 47.52 and AUC of 0.936, followed by Czech Republic, which presents a sensitivity of 0.89, specificity of 0.81, PLR of 4.04, NLR of 0.23, DOR of 16.95 and AUC of 0.930.

Compared with Caucasians, miRNA panels have a higher overall diagnostic accuracy in Asians, with sensitivity of 0.86 versus 0.81, specificity of 0.80 versus 0.76, PLR of 5.91 versus 4.01, NLR of 0.13 versus 0.24, DOR of 44.01 versus 16.73, and AUC of 0.936 versus 0.876, respectively.

Regarding biological specimens, miRNA panels have the highest diagnostic accuracy in serum rather than in other specimens, with a sensitivity, specificity, PLR, NLR, DOR, and AUC of 0.87, 0.86, 7.33, 0.13, 55.29, and 0.943, respectively.

### Sensitivity analysis

Additionally, in order to further reveal the likely origin of heterogeneity, a leave-one-out sensitivity analysis was performed. This study revealed that DOR values did not differ markedly, which indicated that the meta-analysis had strong reliability. Indeed, the DOR values ranged from 18.23 (95% CI 8.42–33.26), *P* < 0.00001 to 23.72 (95% CI 10.82–44.62), *P* < 0.00001 (Table 3).

**TABLE 3:** Sensitivity analyses of diagnostic value of microRNA panels for diagnosis of colorectal cancer in terms of diagnostic odds ratio values.

Study excluded	DOR (95% CI)	Heterogeneity
Chang et al. (30)	20.21 (8.64, 38.97) (*P* < 0.00001)	Chi^2^ = 2077.08 (*P* < 0.00001); *I*^2^ = 99%
Choi et al. (46)	22.94 (10.15, 43.84) (*P* < 0.00001)	Chi^2^ = 2149.91 (*P* < 0.00001); *I*^2^ = 99%
Fang et al. (31)	22.40 (9.76, 43.15) (*P* < 0.00001)	Chi^2^ = 2179.48 (*P* < 0.00001); *I*^2^ = 99%
Guo et al. (32)	19.71 (8.50, 37.71) (*P* < 0.00001)	Chi^2^ = 1997.04 (*P* < 0.00001); *I*^2^ = 99%
Han et al. (33)	18.23 (8.42, 33.26) (*P* < 0.00001)	Chi^2^ = 1621.96 (*P* < 0.00001); *I*^2^ = 99%
Herreros-Villanueva et al. (42)	21.13 (9.01, 40.97) (*P* < 0.00001)	Chi^2^ = 2166.06 (*P* < 0.00001); *I*^2^ = 99%
Huang et al. (34)	20.80 (8.86, 40.30) (*P* < 0.00001)	Chi^2^ = 2142.37 (*P* < 0.00001); *I*^2^ = 99%
Jin et al. (35)	22.12 (9.56, 42.72) (*P* < 0.00001)	Chi^2^ = 2187.12 (*P* < 0.00001); *I*^2^ = 99%
Kanaan et al. (44)	23.40 (10.53, 44.33) (*P* < 0.00001)	Chi^2^ = 2108.89 (*P* < 0.00001); *I*^2^ = 99%
Krawczyk et al. (47)	25.07 (12.23, 45.30) (*P* < 0.00001)	Chi^2^ = 1848.07 (*P* < 0.00001); *I*^2^ = 99%
Liu et al. (40)	20.91 (8.91, 40.53) (*P* < 0.00001)	Chi^2^ = 2151.12 (*P* < 0.00001); *I*^2^ = 99%
Peng et al. (36)	23.22 (10.38, 44.15) (*P* < 0.00001)	Chi^2^ = 2126.14 (*P* < 0.00001); *I*^2^ = 99%
Rapado-González et al. (43)	25.11 (12.28, 45.31) (*P* < 0.00001)	Chi^2^ = 1839.60 (*P* < 0.00001); *I*^2^ = 99%
Tan et al. (37)	19.35 (8.43, 36.72) (*P* < 0.00001)	Chi^2^ = 1925.31 (*P* < 0.00001); *I*^2^ = 99%
Vychytilova-Faltejskova et al. (48)	21.76 (9.35, 32.14) (*P* < 0.00001)	Chi^2^ = 2188.18 (*P* < 0.00001); *I*^2^ = 99%
Wang et al. (41)	23.72 (10.82, 44.62) (*P* < 0.00001)	Chi^2^ = 2071.41 (*P* < 0.00001); *I*^2^ = 99%
Wang et al. (38)	21.66 (9.29, 41.98) (*P* < 0.00001)	Chi^2^ = 2186.82 (*P* < 0.00001); *I*^2^ = 99%
Wikberg et al. (49)	22.98 (10.18, 43.89) (*P* < 0.00001)	Chi^2^ = 2146.82 (*P* < 0.00001); *I*^2^ = 99%
Wu et al. (45)	21.66 (9.29, 41.98) (*P* < 0.00001)	Chi^2^ = 2186.82 (*P* < 0.00001); *I*^2^ = 99%
Zhang et al. (39)	22.55 (9.86, 43.35) (*P* < 0.00001)	Chi^2^ = 2173.34 (*P* < 0.00001); *I*^2^ = 99%

### Publication bias

**FIGURE 6:**
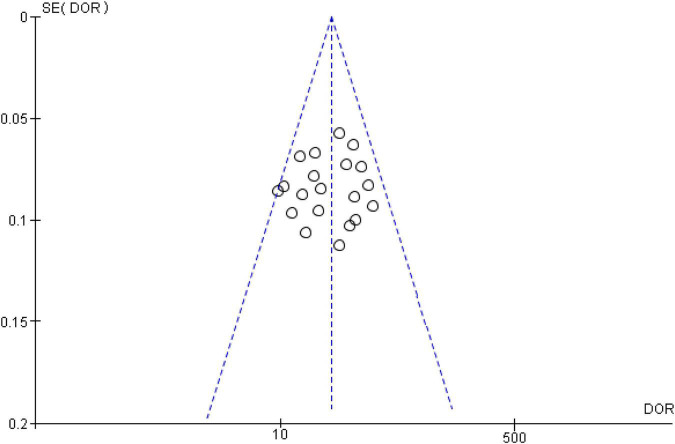
Funnel plot showing no publication bias in terms of diagnostic odds ratio values among the included.

To assess potential publication bias of included studies, the funnel plot asymmetry test was conducted. This study demonstrated no proof of publication bias for DOR values analyzed using Egger’s regression test (*P* > 0.05). Moreover, visual inspection of the funnel plot revealed a symmetrical funnel (Figure 6).

## Discussion

One of the most significant reasons for CRC incidence and its progression is epigenetic modification, which includes differential miRNA expression (50). Recent research has revealed that several classes of miRNAs can be used as biomarkers for CRC diagnosis. The clinical utility of miRNAs as effective biomarkers includes accurate diagnostic value, stable presence in human fluids, and non-invasive detection (51). Hence, this study conducted such a systematic review and meta-analysis which is, to the best of our knowledge, the first to assess the effectiveness of miRNA panels for CRC diagnosis. This systematic review and meta-analysis investigated 20 miRNAs panels reported to be dysregulated in 3339 CRC cases and 2,468 healthy controls.

This meta-analysis of 20 articles revealed that miRNA panels maintained high sensitivity (0.85) and specificity (0.79) in CRC diagnosis. Pooled PLR was 4.06, indicating that the probability of CRC increased by 4.06-folds with positive miRNA panels testing. Moreover, NLR was 0.20, implying that the probability of CRC increased by 80% when the studied miRNA panels were negative. Although a DOR of 1 suggests miRNAs failed to differentiate CRC and control, the DOR of 22.50 in our study showed that miRNAs are outstanding biomarkers in CRC diagnosis. The AUC value is an effective indicator for the assessment system. An ideal test with perfect discrimination is at an AUC of 1.0. As the AUC value of a test approaches 1.0, the overall efficacy of the test will increase. Here, this study found that miRNA panels could be used to screen CRC patients compared to healthy controls, with an AUC value of 0.915, which is very close to 1.0. This suggests that miRNAs have a relatively high ability to distinguish CRC patients from healthy humans.

Assisting clinical decision-making is the most important value of biomarkers. Likelihood ratios are helpful for clinicians since they supply information regarding the likelihood that a patient with a positive or negative test actually has CRC or not. This study also summarized positive likelihood ratios and negative likelihood ratios to judge the clinical applicability of miRNAs for diagnosis. PLR > 10 and NLR < 0.1 represent a high diagnostic accuracy (52). This study found that miRNA panels from articles by Han et al., Tan et al., and Guo et al. had high diagnostic accuracy and clinical applicability (32, 33, 37). Hence, the panel of miRNAs such as (miR15b, miR-21, miR-31), (miR-144-3p, miR-425-5p, miR-1260b) and (miRNA-1246, miRNA-202-3p, miRNA-21-3p, miRNA-1229-3p, miRNA-532-3p) can be deemed promising miRNAs and deserve future research.

Several studies have investigated the diagnostic potential of single miRNAs in CRC. Indeed, miR-21* (32, 33, 35, 49), miR-92a (30, 34, 46), miR-18a (39, 42, 49), miR-144* (37, 45, 46), and miR-29a (34, 42, 43), which were the most studied miRNAs in this meta-analysis, were identified as possible non-invasive biomarkers for CRC. MiR-21-5p can regulate a variety of target genes and pathways involved in tumor growth, invasion, and metastasis, as well as play a significant role in 5-FU resistance and CRC cell radiosensitivity (53–55). Zhang et al. demonstrated that miR-425-5p can influence chemoresistance in CRC cells through modulating programmed cell death (56). However, the cause of the miRNA dysregulation is not always known. MiRNAs that are consistently downregulated or upregulated only in the presence of disease should also be considered as reliable biomarkers. The panel proposed by Han et al. (33), consisting of miR-15b, miR-21, and miR-31, distinguished itself by having the highest sensitivity and specificity in discriminating the healthy control group from the CRC group.

Recently, Zuo et al. conducted a meta-analysis investigating the diagnostic accuracy of single miRNAs in CRC patients (57). In this meta-analysis, including 33 articles published between 2009 and 2019, Zuo et al. showed that the sensitivity, specificity, PLR, NLR, DOR, and AUC of overall miRNAs were 0.80, 0.80, 4.00, 0.26, 16, and 0.87, respectively, which were similar to this study’s results. Moreover, they revealed that serum-derived miRNAs differentiated CRC patients from controls with the highest accuracy, when compared with other biological specimens, which is in line with our findings and indicating that sample types might play a critical role in investigating the utility of miRNAs as biomarkers in disease diagnosis. While Zuo et al. showed no significant difference in diagnostic value between Asian and Caucasian populations, this study revealed that Asians showed higher diagnostic value of miRNA panels than Caucasians, implying that expression difference of miRNA panels within differing ethnicities can also influence diagnostic value of miRNA panels. Furthermore, this meta-regression and subgroup analysis found other sources of heterogeneity, suggesting that ethnicity, country, and biological specimens influence the final results. However, there is almost no publication bias by the funnel plot asymmetry test, strengthening the reliability of the abovementioned findings.

### Strengths and limitations

This study performed a literature search across five differing databases. The principal strong item of this article was the considerable scale of studies and the high number of participants analyzed. Although unpublished articles were not included in this study, the funnel plot did not show a publication bias. The major strength of this meta-analysis was the high methodological quality of the included studies, which presented a low risk of bias. Furthermore, all studies used identical measurement methods, ensuring an effective assessment of miRNA levels when comparing results of differing studies, and consequently renders pooled analysis more reliable.

Although this meta-analysis has offered a systematic and scientific evaluation of miRNA panels for the diagnosis of CRC, some limitations must be noted. Firstly, there were diverse miRNA panels evaluated across the included studies. Consequently, it is difficult to adequately assess the effectiveness of each panel and compare results between studies. Hence, it would be beneficial for future studies to follow a more standardized approach. Secondly, the studies showed strong heterogeneity in the number of subjects included, the biological specimens, and the ethnic populations studied. These differences caused major difficulties to synthesize all available studies. Hence, considerable heterogeneity, which is expected in meta-analysis studies, can alter interpretability of results (58). Consequently, the findings of the present work have to be analyzed with attentiveness. Finally, this study only included studies written in English, which could have affected study findings.

## Conclusion

Our findings showed that miRNA panels had a reasonably good diagnostic biomarker capacity for CRC. Specifically, miR-15b, miR-21 and miR-31 presented the best diagnostic accuracy values for CRC. Thus, they could be used as potential biomarkers for CRC identification within clinical settings. However, large-scale and good quality clinical trials should be conducted to verify our findings and confirm the clinical value of miRNAs in CRC patients in additional details.

## Data availability statement

The raw data supporting the conclusions of this article will be made available by the authors, without undue reservation.

## Author contributions

DS and SA: conception and design of the work, literature review, and data collection. SA and DB: critical revision of the manuscript. All authors drafting the article and have read and agreed to the current version of the manuscript.
